# *Lavandula pedunculata* (Mill.) Cav. Aqueous Extract Antibacterial Activity Improved by the Addition of *Salvia rosmarinus* Spenn., *Salvia lavandulifolia* Vahl and *Origanum compactum* Benth

**DOI:** 10.3390/life12030328

**Published:** 2022-02-22

**Authors:** Salima Boutahiri, Bruno Eto, Mohamed Bouhrim, Hamza Mechchate, Asmaa Saleh, Omkulthom Al kamaly, Aziz Drioiche, Firdaous Remok, Jennifer Samaillie, Christel Neut, Bernard Gressier, Ferdinand Kouoh Elombo, Laila Nassiri, Touriya Zair, Sevser Sahpaz

**Affiliations:** 1 Univ. Lille, University of Liège, University of Picardie Jules Verne, JUNIA, UMRT 1158 BioEcoAgro, Specialized Metabolites of Plant Origin, F-59000 Lille, France; salima.boutahiri.etu@univ-lille.fr (S.B.); jennifer.samaillie@univ-lille.fr (J.S.); sevser.sahpaz@univ-lille.fr (S.S.); 2 Research Team of Chemistry of Bioactive Molecules and the Environment, Laboratory of Innovative Materials and Biotechnology of Natural Resources, Faculty of Sciences, Moulay Ismaïl University, B.P. 11201 Zitoune, Meknes 50070, Morocco; mohamed.bouhrim@gmail.com (M.B.); a.drioiche@edu.umi.ac.ma (A.D.); f.remok@edu.umi.ac.ma (F.R.); t.zair@umi.ac.ma (T.Z.); 3 Laboratoires TBC, Laboratory of Pharmacology, Pharmacokinetics and Clinical Pharmacy, Faculty of Pharmacy, University of Lille, 3, Rue du Professeur Laguesse, B.P. 83, F-59000 Lille, France; bruno.eto@univ-lille.fr (B.E.); ferdinand.kouoh-elombo@univ-lille.fr (F.K.E.); 4 Laboratory of Inorganic Chemistry, Department of Chemistry, University of Helsinki, P.O. Box 55, FI-00014 Helsinki, Finland; 5 Department of Pharmaceutical Sciences, College of Pharmacy, Princess Nourah bint Abdulrahman University, P.O. Box 84428, Riyadh 11671, Saudi Arabia; asali@pnu.edu.ss; 6 U1286 INFINITE Inst Translat Res Inflammat, University of Lille, Inserm, CHU Lille, F-59000 Lille, France; christel.neut@univ-lille.fr; 7 Laboratory of Pharmacology, Pharmacokinetics and Clinical Pharmacy, Faculty of Pharmacy, University of Lille, 3, rue du Professeur Laguesse, B.P. 83, F-59000 Lille, France; bernard.gressier@univ-lille.fr; 8 Research Team of Environment and Valorization of Plant and Microbial Resources, Faculty of Sciences, Moulay Ismaïl University, Meknes, B.P. 11201 Zitoune, Meknes 50070, Morocco; l.nassiri@umi.ac.ma

**Keywords:** *Lavandula pedunculata* (Mill.) Cav., *Salviarosmarinus* Spenn., *Salvia lavandulifolia* Vahl., *Origanum compactum* Benth., polyphenols, antibacterial activity

## Abstract

Lavender aqueous extracts are widely used in the Moroccan traditional medicine for their antibacterial properties. However, previous research have generally focused on investigating the antibacterial activity of lavender essential oils. The aim of this study is to evaluate the antibacterial activity of the Moroccan *Lavandula pedunculata* (Mill.) Cav. aqueous extract, alone, as well as in combination with extracts of other plant species known for their antibacterial activity: *Salvia rosmarinus* Spenn., *Salvia lavandulifolia* Vahl. and *Origanum compactum* Benth. We have tested the antibacterial activity of *L. pedunculata*, *S. rosmarinus*, *S. lavandulifolia* and *O. compactum* aqueous extracts individually and in combination against 34 strains using the agar dilution method. The combination effect was evaluated using the fractional inhibitory concentration (FIC). Polyphenol and tannin contents were determined using Folin-Ciocalteu reagent, and then some phenolic compounds were identified using UHPLC-MS. All the extracts displayed a large spectrum of antibacterial activity, especially against staphylococci, streptococci, *Mycobacterium smegmatis* and *Proteus mirabilis*. The minimum inhibitory concentration (MIC) values reached 0.15 ± 0.00 mg/mL for *Staphylococcus warneri* tested with *S. lavandulifolia* and 0.20 ± 0.07 mg/mL for *Staphylococcus epidermidis* tested with *L. pedunculata* or *S. rosmarinus*. Association of the *L. pedunculata* extract with *S. rosmarinus*, *S. lavandulifolia* and *O. compactum* showed synergistic effects (FIC ≤ 1). Moreover, the association of *L. pedunculata* with *S. lavandulifolia* was active against most of the Gram-negative strains resistant to the individual extracts. Determination of polyphenol and tannin contents showed the richness of the studied plants in these compounds. Additionally, chromatographic analysis demonstrated the high presence of rosmarinic acid in all the studied plant extracts. To our knowledge, this is the first study that shows the enhancing effect of the antibacterial activity of *L. pedunculata* aqueous extract combined with *S. rosmarinus*, *S. lavandulifolia* and *O. compactum*. These results confirm the effectiveness of the plant mixtures commonly used by traditional healers in Morocco and suggest that *L. pedunculata* might be used as an antibacterial agent either alone or, more efficiently, in combination with *S. rosmarinus*, *S. lavandulifolia* and *O. compactum*.

## 1. Introduction

Infections can cause moderate to severe damages and are a burden to global economies and public health [1]. These infections are increasing due to the emergence of higher virulence, such as the multidrug-resistant bacteria caused by multiple antibiotic treatments [2,3]. Some of the most problematic multidrug resistant organisms that are encountered currently include *Pseudomonas aeruginosa*, *Acinetobacter baumannii*, extended-spectrum beta-lactamases (ESBL)-producing *Escherichia coli* and *Klebsiella pneumoniae*, vancomycin-resistant enterococci, methicillin-resistant *Staphylococcus aureus* and extensively drug-resistant *Mycobacterium tuberculosis* [2,4]. In addition to increasing resistance to existing agents, there is a lack of new antibiotics in development [5].

In order to find solutions for this important issue, more and more research is now made on medicinal plants trying to find new natural sources of antibiotics. Indeed, traditional phytotherapy has been the main source of remedy for various diseases in the past, and several studies have proved its efficacy. Researchers in this field are giving importance to the use of aromatic and medicinal plants as alternatives to antibiotics or as therapeutic complements. Because of their multiple modes of action, plant extracts could be as efficient as antibiotics, with lower risks of causing resistance or side effects. Plants contain several active compounds (secondary metabolites) belonging mainly to three classes: phenolic compound, terpenes and alkaloids [6,7]. In Morocco, 4200 plant species were identified, of which 600 are recognized for their aromatic and medicinal properties [8].

Among aromatic and medicinal plants, lavender is famous for the quality of its essential oils and aqueous extracts that have long been used in traditional medicine, perfumes, cosmetics, hygiene products, food industry and pharmacy [9,10]. In Morocco, lavender is one of the most used plants by the population for the treatment of several diseases [11,12]. Researchers have been investigating its properties and have proven its antidiabetic, antirheumatic, anti-inflammatory, antioxidant, antibacterial, antifungal, antidepressant and antispasmodic activities. It is also efficient in the treatment of respiratory and digestive diseases [13,14,15,16,17,18,19,20]. However, lavender is usually investigated for the activity of its essential oils, regardless of the large use of its aqueous extracts in traditional medicine. Moreover, Moroccan people tend to use lavender in combination with other plants for better results [12,21,22].

*L. pedunculata* is one of the lavender species widely used in traditional Moroccan medicine to treat several diseases [12,21]. However, few research studies have been performed to investigate and prove its efficacy. For these reasons, the aim of this study is to investigate the antibacterial activity of the aqueous extract of Moroccan *L. pedunculata* towards several microbial strains. Moreover, its combinations with *S. rosmarinus*, *S. lavandulifolia* and *O. compactum* from Morocco, known for their antibacterial activities [23,24,25], will also be tested. In addition, the chemical composition of the aqueous extracts of all the chosen plants will be determined using UHPLC-MS.

## 2. Materials and Methods

### 2.1. Chemicals and Reagents

The used standards are luteolin, herniarin and myricetin (purchased from Sarsyntex, Merignac, France), apigenin (obtained from Carl Roth, Karlsruhe, Germany), coumarin (Behringer, Willich, Germany), cinnamic acid (Rhône-Poulenc, Paris, France), protocatechuic acid (Koch-Light Laboratories LTD, Bucks, UK), vanillic acid (Merck, Darmstadt, Germany), chlorogenic acid (Sigma-Aldrich, St. Louis, MO, USA), rosmarinic acid (Extrasynthèse, Genay, France) and gallic acid (Prolabo, Paris, France), as well as ferulic acid and caffeic acid purchased from Sigma (St. Louis, MO, USA). Formic acid (Carlo Erba Reagents^TM^, Cornaredo, Italy) and methanol (Carlo Erba Reagents, Val-de-Reuil, France) were of HPLC grades. All other chemicals used were also of analytical grade.

### 2.2. Plant Material

*L. pedunculata*, *S. rosmarinus*, *S. lavandulifolia* and *O. compactum* were collected from different regions in Morocco (Table 1). The used organs were chosen according to the bibliographic data [22,23,24,26], and they were dried for thirteen days in the open air and in the shade.

Plant identification was carried out at the Scientific Institute of Rabat (Rabat, Morocco) by Dr. Hamid Khamar.

### 2.3. Aqueous Extraction

*L. pedunculata* flowering tops, *S. rosmarinus* leaves, *S. lavandulifolia* leaves and *O. compactum* leaves and flowers were each mixed and heated with distilled water (1:20; *w*/*v*) for one hour at 75 ± 2 °C using a hot plate. The temperature was monitored using an electronic laboratory thermometer. The mixtures were then filtered, and the obtained filtrates were dried in the oven at 70 °C until obtaining dry extract powders. The latter were put in closed flasks away from light and humidity until further use.

### 2.4. Determination of Total Polyphenol Content

Total polyphenol concentrations in the different plant aqueous extracts were determined by the method described by Zhang and his collaborators using the Folin-Ciocalteu reagent with some modifications [27]. In a 96-well microplate, 25 μL of the extract at 0.5 mg/mL were introduced, then 125 μL of the Folin-Ciocalteu reagent (10%) were added, followed by 100 μL of sodium carbonate at 145 mg/mL. After 5 min of orbital stirring and 2 h incubation in the dark at 25 °C, the reading was made at 760 nm by a SPECTROstarNano spectrophotometer. The negative control was prepared according to the same protocol using 25 µL of distilled water instead of the extract.

A standard calibration curve was made from different concentrations of gallic acid. It was used for the calculation of polyphenol concentrations in the extracts. The total phenolic content is therefore expressed in milligrams equivalent of gallic acid per gram of the dry extract (mg GAE/gExt).

### 2.5. Determination of Total Tannin Content

Total tannin concentrations in the aqueous extracts were determined using the hide-powder method. It consists of the determination of polyphenol contents in the extracts after their contact with the hide-powder, which precipitates tannins [28].

1 mL of the 0.5 mg/mL extract was stirred with 10 mg of the hide-powder for 1 h. The mixture was then centrifuged at 2500 rpm for 5 min. The polyphenol content in the supernatant was then determined using the method described above (Section 2.4). The total tannin content in an extract corresponds, therefore, to the difference between the total polyphenol content in the extract and the polyphenol content after precipitating tannins with the hide-powder. This content is expressed in mg GAE/gExt.

### 2.6. UHPLC Analysis of Aqueous Extracts

Chromatographic analysis of the aqueous extracts was carried out on an AQUITY UPLC H-Class System (Waters Corporation, Manchester, UK) equipped with two independent pumps, an automatic injector, a controller, a diode array UV detector (DAD), a mass spectrometer with ESI ionization source and a quadrupole as an analyzer. The stationary phase is a reverse phase Waters^®^ Acquity BEH C18 column (2.1 × 50 mm, 1.7 µm) connected to a 0.2 µm in-line filter. The mobile phase is composed of two solvents: (A) ultrapure water (Milli-Q^®^ Integral 5, Merck^TM^, Darmstadt, Germany) + 0.1% formic acid; (B) methanol + 0.1% formic acid. The elution gradient established was 0–5% B (1 min), 5–20% B (0.5 min), 20% B (3.5 min), 20–100% B (4 min), rinsing of the column 100% B (2 min) and re-equilibration 100–0% B (0.5 min), 0% B (2.5 min). Methanol (70%) was required for washing the system.

The aqueous extracts were solubilized in a methanol/water mixture (1:1, *v*/*v*) to obtain a concentration of 1 mg/mL, and then they were filtered through 0.2 μm PTFE filter. For each analysis, 0.004 mL of the extract was injected. The temperature was set at 30 °C, and the flow rate was set at 0.3 mL/min.

This analysis was carried out on few standards chosen according to bibliographic data. They were injected under the same conditions as those of the extracts. These standards are cinnamic acid, luteolin, apigenin, myricetin, ferulic acid, protocatechuic acid, vanillic acid, chlorogenic acid, caffeic acid, rosmarinic acid, gallic acid, herniarin and coumarin.

Chemical compounds of the extracts were identified by matching their retention time, UV spectrum and molecular weight to those of the used standards.

### 2.7. Antibacterial Activity of Aqueous Extracts

#### 2.7.1. Preparation of Bacterial Suspensions

The chosen microorganisms, some of which are resistant to antibiotics, are involved in various opportunistic or nosocomial infections. They were cultured from suspensions of the strains contained in a liquid Brain Heart Infusion Agar medium in tubes containing a sloping Mueller-Hinton Agar (MHA) culture medium (MHB Oxoid^TM^, Basingstoke, UK; Bacto^TM^ Agar, Le Pont de Claix, France). The latter were incubated for 24 h at 37 °C, and then 10 mL of Ringer Cysteine (RC) liquid (Merck^TM^, Darmstadt, Germany) was added into the tubes. A good mixing is necessary in order to suspend the cultured microorganisms. A drop of each suspension was collected to be added in a dilution tube containing 10 mL of RC solution. The turbidity of the obtained suspension that was used for the test was therefore estimated at 0.5 McFarland. An amount of 1 mL of suspension from each dilution tube was withdrawn to fill the wells of the inoculum replicator plate.

#### 2.7.2. Activity of Individual Extracts

Antibacterial activity of the plants aqueous extracts was evaluated using the agar dilution method [29]. This method allows for determining the MIC of each extract towards 34 microorganisms in in vitro culture. The aqueous extracts were primarily dissolved in water/ethanol (7:3), and then they were mixed with MHA in Petri dishes. The final concentrations tested were 1.2, 0.6, 0.3, 0.15 and 0.075 mg/mL. The Petri dishes containing the MHA-extract mixture were inoculated with the microorganisms using an inoculum replicator, and they were incubated for 24 h at 37 °C. Activity was assessed visually by the presence or absence of culture. MIC values were recorded as the lowest concentrations of extracts that completely inhibit the growth of a specific microbe. A negative control was tested using the solvent. Three antibiotics were used as positive controls: gentamicin, vancomycin and amoxicillin. The studied strains are considered to be susceptible to the used antibiotics when MIC ≤ 4 mg/L, and they are considered to be resistant to gentamicin when MIC > 8 mg/L and resistant to vancomycin and amoxicillin when MIC > 16 mg/L [29,30].

#### 2.7.3. Activity of Extracts in Mixtures

In order to investigate the antibacterial activity of extracts mixtures, the checkerboard assay was used. This method consists in studying all the possible combinations in the range of the chosen concentrations (1.2, 0.6, 0.3, 0.15 and 0.075 mg/mL).

The MIC of an extract mixture is also determined by the agar dilution procedure. It corresponds to the lowest concentrations of mixtures inhibiting the growth of a microbe.

In order to determine the effect of a combination, the FIC is calculated. It is an indicator of the activity of a plant mixture against microbial strains [31]. It is calculated as follows for the combination of extracts A and B:

(1) FIC=∑FIC(X)=FIC(A)+FIC(B)

(2) $$\mathrm{FIC}\left(A\right)=\frac{\mathrm{MIC}\;\mathrm{of}\;A\;\mathrm{in}\;\mathrm{combination}}{\mathrm{MIC}\;\mathrm{of}\;A\;\mathrm{alone}}$$

(3) $$\mathrm{FIC}\left(B\right)=\frac{\mathrm{MIC}\;\mathrm{of}\;B\;\mathrm{in}\;\mathrm{combination}}{\mathrm{MIC}\;\mathrm{of}\;B\;\mathrm{alone}}$$

FIC value tells the effect of the combination:

- FIC < 1: synergistic effect;
- FIC = 1: commutative effect;
- 1 < FIC ≤ 2: indifferent effect;
- 2 < FIC: antagonistic effect.

### 2.8. Statistical Analysis

Data are presented as means ± standard deviations. Their statistical analysis was performed using GraphPad Prism version 5.00 for Windows, GraphPad Software, San Diego, California, USA. Multiple-group comparisons were analyzed using the one-way analysis of variance (ANOVA). Statistical significance was defined as *p* < 0.05.

## 3. Results

### 3.1. Determination of Total Polyphenol and Total Tannin Contents

**Figure 1:**
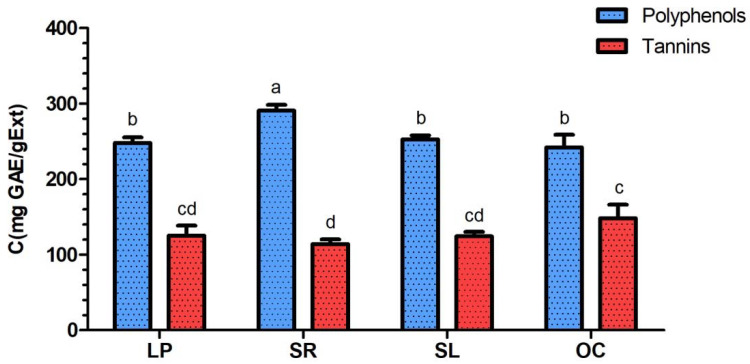
Total polyphenol and tannin contents in *L. pedunculata* (LP), *S. rosmarinus* (SR), *S. lavandulifolia* (SL) and *O. compactum* (OC) aqueous extracts. Contents with common letters (a, b, c and d) are not significantly different.

Polyphenol and tannin contents in *L. pedunculata*, *S. rosmarinus*, *S. lavandulifolia* and *O. compactum* aqueous extracts are shown in Figure 1. The highest polyphenol content was found in *S. rosmarinus* (290.63 ± 7.69 mg GAE/gExt), and it is significantly (*p* < 0.01) different from the three other contents, which are 248.05 ± 7.27, 252.67 ± 5.40 and 241.90 ± 16.95 mg GAE/gExt, respectively, for *L. pedunculata*, *S. lavandulifolia* and *O. compactum*.

As for tannin contents, *O. compactum* extract (148.20 ± 17.96 mg GAE/gExt) was significantly (*p* < 0.05) richer in tannins than *S. rosmarinus* (113.93 ± 6.16 mg GAE/gExt), but both extracts (*O. compactum* and *S. rosmarinus*) do not significantly differ from *L. pedunculata* (125.13 ± 13.26 mg GAE/gExt) and *S. lavandulifolia* (124.25 ± 6.07 mg GAE/gExt).

### 3.2. UHPLC Analysis of Aqueous Extracts

**Figure 2:**
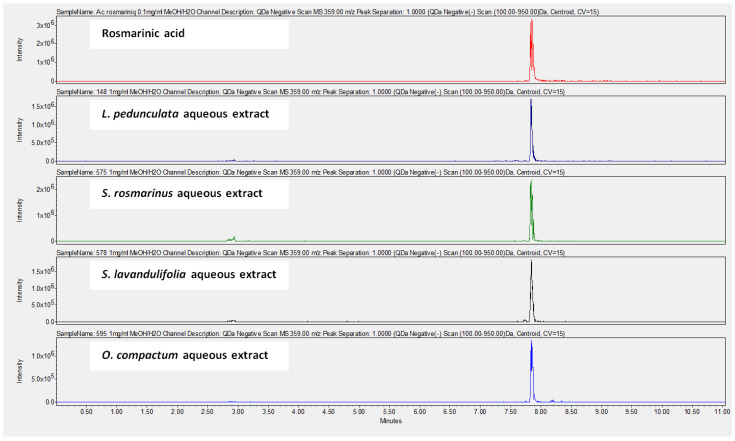
UHPLC-MS chromatograms at 359.00 *m*/*z* (negative scan) showing the presence of rosmarinic acid in *L. pedunculata*, *S. rosmarinus*, *S. lavandulifolia* and *O. compactum* aqueous extracts.

The chemical composition of the aqueous extracts was determined using UHPLC-MS. The results (Table 2) show that the most abundant compound in all the studied extracts is rosmarinic acid. Figure 2 shows the UHPLC-MS chromatograms of rosmarinic acid detected in the aqueous extracts.

Moreover, coumarin, apigenin, luteolin, protocatechuic acid, vanillic acid, gallic acid and caffeic acid are found in the different extracts but with a lower abundance or as traces. We can notice that herniarin and myricetin are present in all the plants except *S. rosmarinus*. As for ferulic acid, it is present only in *L. pedunculata* and *S. rosmarinus* extracts. Cinnamic acid is absent in *O. compactum* extract, and chlorogenic acid is absent in *L. pedunculata*.

### 3.3. Antibacterial Activity of Aqueous Extracts

**Figure 3:**
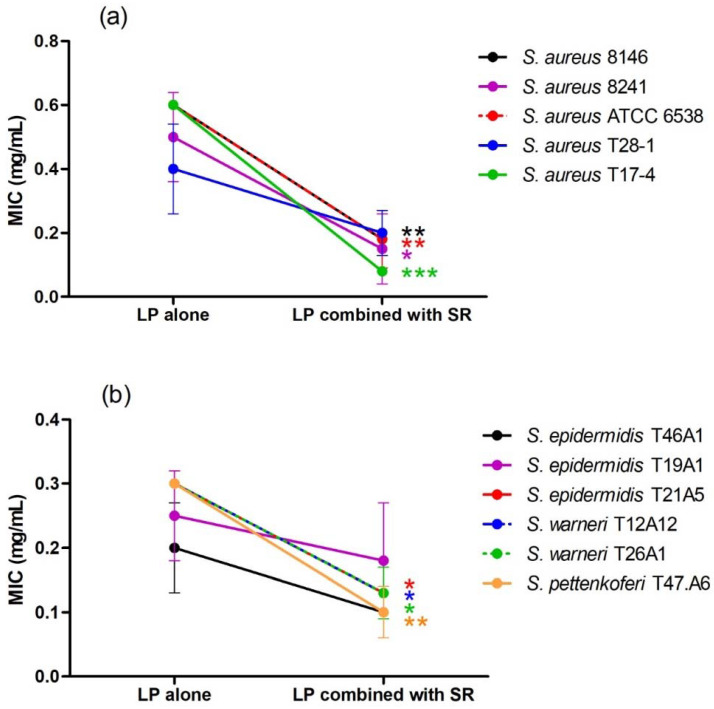
Effect of the association of *L. pedunculata* (LP) with *S. rosmarinus* (SR) on MIC values of LP ((**a**) = *S. aureus* strains, (**b**) = coagulase negative staphylococci). After combination, a remarkable improvement of the antibacterial activity was observed. MIC values of LP combined with SR are significantly lower than MIC values of LP alone * *p* < 0.05; ** *p* < 0.01; *** *p* < 0.001.

**Figure 4:**
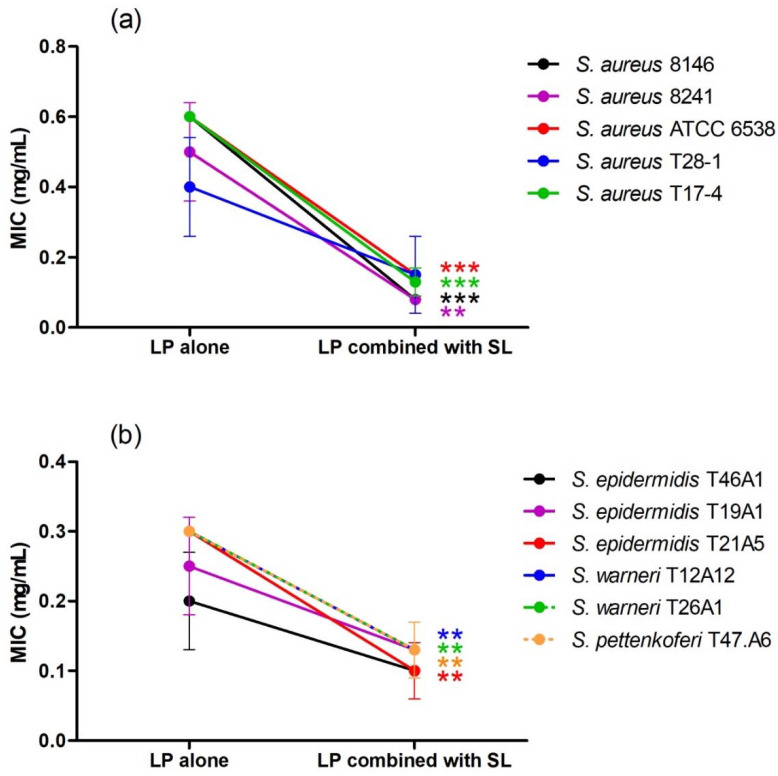
Effect of the association of *L. pedunculata* (LP) with *S. lavandulifolia* (SL) on MIC values of LP ((**a**) = *S. aureus* strains, (**b**) = coagulase negative staphylococci). After combination, MIC values of LP combined with SL are significantly lower than MIC values of LP alone ** *p* < 0.01: *** *p* < 0.001.

**Figure 5:**
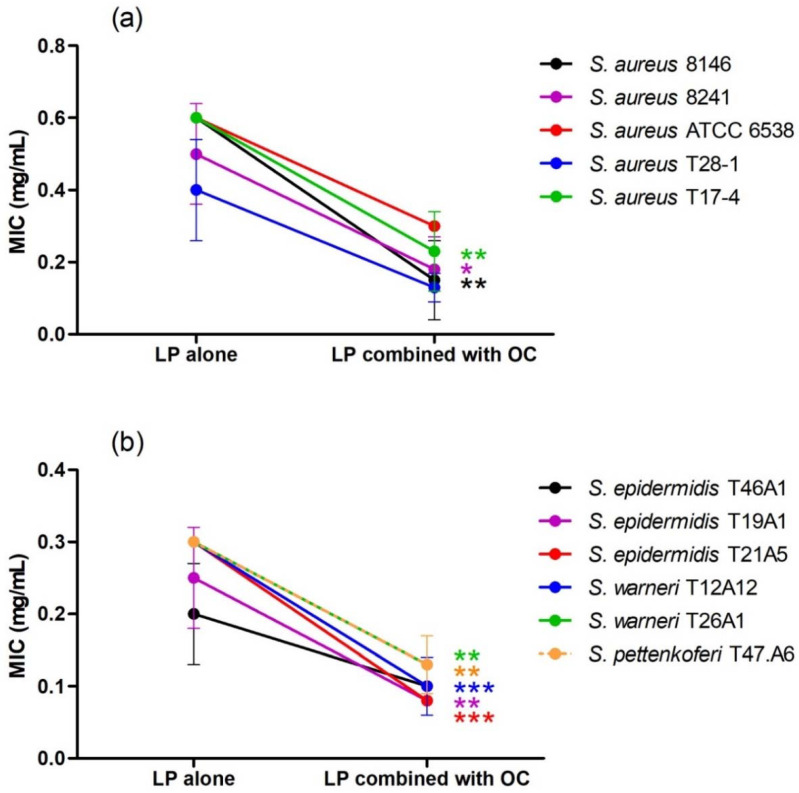
Effect of the association of *L. pedunculata* (LP) with *O. compactum* (OC) on MIC values of LP ((**a)** = *S. aureus* strains, (**b**) = coagulase negative staphylococci). After combination, MIC values of LP combined with OC are significantly lower than MIC values of LP alone * *p* < 0.05; ** *p* < 0.01: *** *p* < 0.001.

*L. pedunculata*, *S. rosmarinus*, *S. lavandulifolia* and *O. compactum* aqueous extracts were tested individually and in association against 34 bacteria. Moreover, different antibiotics (gentamicin, vancomycin and amoxicillin) were tested against the same bacteria. The different results of their antibacterial activities are shown in Table 3, Table 4, Table 5 and Table 6 and Figure 3, Figure 4 and Figure 5.

All the individual extracts are active against staphylococci and streptococci, as well as against *Proteus mirabilis*, *Mycobacterium smegmatis* and *Pseudomonas aeruginosa*. *L. pedunculata* extract is also active against one of the two tested strains of *Citrobacter freundii*. The lowest MIC obtained after using *L. pedunculata* and *S. rosmarinus* extracts was 0.20 ± 0.07 mg/mL against *S. epidermidis* T46A1 (Table 4), and 0.35 ± 0.19 mg/mL is the lowest MIC obtained by *O. compactum* against the same strain (Table 6). As for *S. lavandulifolia*, the lowest MIC value is 0.15 ± 0.00 mg/mL, and it is obtained against *S. warneri* T26A1 (Table 5).

After combining *L. pedunculata* extract with *S. rosmarinus*, *S. lavandulifolia* and *O. compactum*, a remarkable improvement of the antibacterial activity was observed. On one hand, additive and synergistic effects were noticed on the majority of the strains that are susceptible to the individual extracts. On the other hand, we noticed that extract mixtures were active against several strains that are resistant to the individual extracts, especially Gram-negative strains such as *E. coli*, *E. aerogenes*, *K. pneumoniae* and *Salmonella* sp. Moreover, the association of *L. pedunculata* with *S. lavandulifolia* was active against 32 strains among 33 (the strain *S. agalactiae* T38.2 was not tested because of some experimental issues), and it was the most effective combination. The association of *L. pedunculata* with *S. rosmarinus* came in the second position by being active against 30 strains, followed by the mixture of *L. pedunculata* with *O. compactum* that was active against 25 strains. It is remarkable that some plant mixtures were active against the majority of the Gram-negative strains often multidrug-resistant (Table 3).

## 4. Discussion

Lavender species are widely used for their antibacterial activity demonstrated in several studies [32,33]. However, their essential oils are usually studied regardless of the large use of their aqueous extracts in the traditional medicine [34,35,36]. Besides, the use of essential oil is very limited in human and animal pharmacy due to the potential occurrence of side effects and toxicity [37].

In the present study, we tested the antibacterial effect of *L. pedunculata* aqueous extract that showed a strong antibacterial activity mainly against Gram-positive strains. Lopes and his collaborators investigated the antibacterial activity of *L. pedunculata* aqueous extract from Portugal using the microdilution method. They found MIC values ranging from 0.10 to 0.45 mg/mL for *E. coli*, and from 0.15 to 0.45 mg/mL for *S. aureus*, *P. aeruginosa* and S. Typhimurium. Some of these data are close to the results of our study, where *L. pedunculata* was active against *S. aureus* with MIC values ranging from 0.4 to 0.6 mg/mL and against *P. aeruginosa* with MIC values ranging from 0.5 to 1.2 mg/mL. However, our extract was not active against *Salmonella* sp. and *E. coli* [26].

In addition to *L. pedunculata*, the aqueous extracts of three other plant species, *S. rosmarinus*, *S. lavandulifolia* and *O. compactum*, were also tested in this study and were active against several bacterial strains, especially the Gram-positive ones. In a study carried out by Ramdan et al. (2018), the antibacterial activity of the hydroethanolic extracts of *S. rosmarinus* and *O. compactum* from Marrakech (Morocco) was evaluated by the broth microdilution method. The respective MIC values obtained for *S. rosmarinus* and *O. compactum* were 25 and 12.5 mg/mL for *Salmonella enterica*, 50 and 25 mg/mL for *E. coli* and *P. aeruginosa*, and 12.5 mg/mL for both extracts against *S. aureus*. These values are very high in comparison with our study [38]. Giner and his collaborators conducted a study on the hydroalcoholic extract mixture of *S. lavandulifolia* with *S. rosmarinus* and *Thymus mastichina*. They showed that it is active against *E. coli* and *E. aerogenes* with a MIC value of 12.8 mg/mL. This mixture was also active against *S. enterica* and *S. aureus*, with respective MIC values of 6.4 and 0.4 mg/mL. These results are very different from the ones obtained in this work, except for *S. aureus* which is quite similar [39].

The three species were not only tested individually, but also in association with *L. pedunculata*, thus giving promising activities that were found for the first time. In fact, some plant mixtures, such as the mixture of *L. pedunculata* with *S. lavandulifolia*, were active against the majority of the Gram-negative strains, often multidrug-resistant, while the individual extracts were not active. Moreover, activities of the plant mixtures against the Gram-positive strains were boosted. One of the most problematic Gram-positive strains was *S. aureus*. It is a human pathogen that possesses a high adaptability and tenacity, making it abundant in the environment. It is capable of colonizing various human organs, and it is a source of a variety of virulence factors. The multidrug-resistant form of this microorganism, especially the methicillin-resistant *S. aureus*, is one of the major microorganisms responsible for bloodstream infections that cause high levels of mortality worldwide. Since *S. aureus* has succeeded in developing resistance against practically all antibiotics, finding a new alternative is urgently needed, of which includes the importance of plant extracts such as the ones tested in this study and that showed strong activities [40,41].

Polyphenol and tannin contents of *L. pedunculata*, *S. rosmarinus*, *S. lavandulifolia* and *O. compactum* aqueous extracts were determined during the present study using the Folin-Ciocalteu method. The highest polyphenol content corresponded to *S. rosmarinus* extract (290.63 ± 7.70 mg GAE/gExt). This result was significantly high while compared to the ethanolic extract of the same plant species from Taiwan that has a polyphenol content of 161.07 ± 3.12 mg GAE/gExt [42]. Moreover, ethanolic extract of *S. rosmarinus* from different regions in Morocco also had lower polyphenol contents ranging from 74.15 to 146.63 mg GAE/gExt [43]. As for *L. pedunculata*, *S. lavandulifolia* and *O. compactum* aqueous extracts studied in the present work, their respective polyphenol contents are 248.03 ± 7.30, 252.67 ± 5.41 and 241.90 ± 16.96 mg GAE/gExt. The polyphenol content of *Salvia officinalis* aqueous extract from Portugal was determined by Afonso et al. (2019), and it is similar to *S. lavandulifolia* studied in this work (229.0 ± 44.0 mg GAE/gExt) [44]. Furthermore, a study was conducted on the ethanolic extracts of *O. compactum* aerial parts from Ouezzane and Taounate (Morocco), and their polyphenol contents were found to be lower than in our extract, with respective values of 117.60 ± 1.12 and 117.56 ± 2.74 mg GAE/gExt [23,45].

As for the tannin contents, the obtained values represent almost the half of the polyphenol contents, with respective values of 125.13 ± 13.24, 113.93 ± 6.15, 124.23 ± 6.08 and 148.20 ± 17.97 mg GAE/gExt for *L. pedunculata*, *S. rosmarinus*, *S. lavandulifolia* and *O. compactum*. Indeed, tannins are known for their antibacterial properties [46,47]; therefore, they might be the compounds that are responsible for the observed activities of the studied plant extracts.

UHPLC analysis of the plant extracts demonstrated their high content in rosmarinic acid. In fact, rosmarinic acid is found to be a good antibacterial agent [48]. This compound has the ability of damaging the cell membrane [49]. Other compounds were also detected in the studied extracts that are known for their antibacterial and antifungal activities such as coumarin, apigenin and caffeic acid [49,50,51].

Lopes et al. (2018) analyzed the phenolic compounds of *L. pedunculata* aqueous extract from 13 different natural populations in Portuguese regions using HPLC-DAD-ESI/MSn. They found that phenolic acids represent the major phenolic compounds present in these extracts. Salvianolic acid B and rosmarinic acid were present in large concentrations and caffeic acid in smaller ones. Concerning flavonoids, the main present compound was luteolin-7-O-glucuronide [26]. In another study also conducted on *L. pedunculata* from Portugal, different extracts were analyzed using HPLC/DAD. The obtained results showed that these extracts contain high concentrations of rosmarinic acid and smaller ones of luteolin. Moreover, apigenin was not quantified in the aqueous extracts, but it was present in the ethanolic and hydroethanolic ones [52]. Some studies conducted on the methanolic and ethanolic extracts of *S. rosmarinus* showed the presence of caffeic acid, rosmarinic acid, vanillic acid and ferulic acid [24,53]. Furthermore, in a review concerning polyphenolic compounds of *Salvia* species, it was mentioned that *S. lavandulifolia* contains rosmarinic acid, apigenin and luteolin [54]. All of these studies are in concordance with the results we found in this research work.

## 5. Conclusions

The results obtained in the present study demonstrated that *L. pedunculata* aqueous extract from Morocco exerts an important antibacterial activity mainly against Gram-positive bacteria. Moreover, this activity is boosted when *L. pedunculata* extract is used in mixtures with *S. rosmarinus*, *S. lavandulifolia* and *O. compactum*. Some active compounds were investigated, and all the extracts were shown to contain high amounts of polyphenols and tannins. These results represent a first step in investigating the use of *L. pedunculata* aqueous extracts by the Moroccan population. Furthermore, these results showed the effectiveness of the alternative and combinative polyphytotherapy. This suggests that *L. pedunculata* aqueous extract could be used as a new potential source of natural antibacterial agents either alone or in combination with *S. rosmarinus*, *S. lavandulifolia* and *O. compactum*. It might be an effective solution for resistant bacteria that cause damage around the world, such as *S. aureus*. However, clinical studied are needed to confirm these activities on real microbial infection cases. Ultimately, this could contribute to the valorization of biodiversity and resources of Morocco and could generate new sources of income for the population.

## Figures and Tables

**Table 1:** Information about the studied plants.

Plant Species	Family	Voucher Number	Used Organ	Harvest Region	Geographic Coordinates	Harvest Date
*Lavandula pedunculata* (Mill.) Cav.	*Lamiaceae*	RAB111854	Flowering tops	Taza	34°04′01.2″ N 4°07′42.5″ W	May 2019
*Salvia rosmarinus* Spenn.	*Lamiaceae*	RAB111855	Leaves	Oulad Ali	33°27′46.6″ N 3°58′34.4″ W	May 2017
*Salvia lavandulifolia* Vahl	*Lamiaceae*	RAB111857	Leaves	Oulad Ali	33°27′45.2″ N 3°58′39.8″ W	May 2017
*Origanum compactum* Benth.	*Lamiaceae*	RAB111858	Leaves and flowers	Bouyablane	33°39′02.4″ N 4°09′49.6″ W	June 2018

**Table 2:** Results of UHPLC-MS analysis regarding the presence or the absence of some chemical compounds in *L. pedunculata*, *S. rosmarinus*, *S. lavandulifolia* and *O. compactum* aqueous extracts.

*t*_R_ (min)	λ_max_ (nm)	[M − H]^−^ (*m/z*)	[M + H]^+^ (*m/z*)	Compounds	LP	SR	SL	OC
6.869	278.1	WD	147	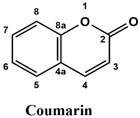	+	T	T	T
8.259	255.5, 297.2	147	149	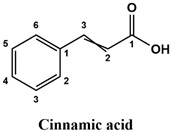	T	+	T	−
2.697	259.1, 293.6	153	155	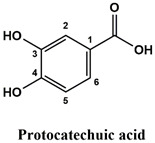	+	+	+	+
3.923	260.3, 292.4	167	169	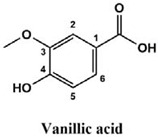	+	+	T	+
1.518	371.0	169	171	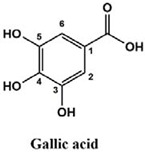	+	T	T	T
7.761	307.9	WD	177	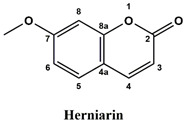	+	−	+	T
3.976	324.7	179	181	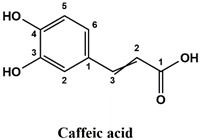	+	+	+	+
6.536	322.3	193	195	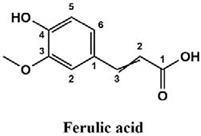	+	+	−	−
8.606	338.8	269	271	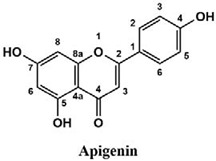	+	+	+	+
8.368	254.3, 350.4	285	287	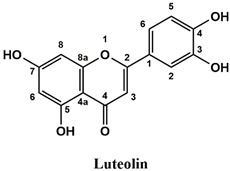	+	+	+	+
7.792	253.2, 372.1	317	319	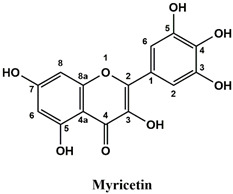	+	−	+	T
3.680	240.1, 325.8	353	355	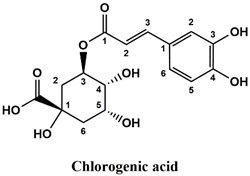	−	T	T	T
7.831	329.4	359	WD	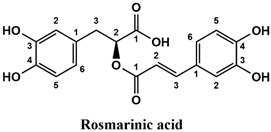	++	++	++	++

(WD) weak detection; (T) traces; (−) absence; (+) presence; (++) high presence; (LP) *L. pedunculata*; (SR) *S. rosmarinus*; (SL) *S. lavandulifolia*; (OC) *O. compactum.*

**Table 3:** MIC of antibiotics (gentamycin, vancomycin and amoxicillin) in mg/L.

Microorganisms		Reference	Antibiotics (MIC Values in mg/L)		
			Gentamicin	Vancomycin	Amoxicillin
Gram +	*Enterococcus faecalis*	C159-6	2	0.5	64
	*Enterococcus* sp.	8153	2	4	2
	*Mycobacterium smegmatis*	5003	0.03	0.5	1
	*Staphylococcus aureus*	8146	0.5	1	4
	*Staphylococcus aureus*	8241	0.5	1	16
	*Staphylococcus aureus*	ATCC 6538	0.25	1	0.125
	*Staphylococcus aureus*	T28-1	0.5	1	2
	*Staphylococcus aureus*	T17-4	0.5	1	1
	*Staphylococcus epidermidis*	T46A1	0.06	2	1
	*Staphylococcus epidermidis*	T19A1	32	2	16
	*Staphylococcus epidermidis*	T21A5	0.06	2	16
	*Staphylococcus warneri*	T12A12	0.06	4	1
	*Staphylococcus warneri*	T26A1	0.06	2	0.25
	*Staphylococcus pettenkoferi*	T47.A6	0.06	2	0.25
	*Streptococcus agalactiae*	T38.2	ND	ND	ND
	*Streptococcus agalactiae*	T53C9	0.5	0.25	0.03
	*Streptococcus pyogenes*	16138	0.125	0.25	0.03
	*Streptococcus pyogenes*	16135	0.125	0.25	0.03
	*Corynebacterium striatum*	T40A3	0.06	0.5	1
Gram −	*Citrobacter freundii*	11041	0.25	NA	2
	*Citrobacter freundii*	10268	ND	ND	ND
	*Escherichia coli*	ATCC 25922	0.5	NA	16
	*Escherichia coli*	T20A1	0.25	NA	NA
	*Escherichia coli*	8138	0.5	NA	NA
	*Escherichia coli*	8157	0.5	NA	NA
	*Enterobacter aerogenes*	9004	0.5	NA	NA
	*Klebsiella pneumoniae*	10270	8	NA	NA
	*Klebsiella pneumoniae*	11016	0.25	NA	NA
	*Proteus mirabilis*	11060	0.5	NA	2
	*Proteus mirabilis*	T28-3	0.25	NA	1
	*Pseudomonas aeruginosa*	8131	1	NA	NA
	*Pseudomonas aeruginosa*	ATCC 27583	2	NA	NA
	*Pseudomonas aeruginosa*	8129	0.03	NA	NA
	*Salmonella* sp.	11033	0.25	NA	2

(ND) not determined; (NA) Not active.

**Table 4:** MIC values of *L. pedunculata* and *S. rosmarinus* aqueous extracts individually and in combination (in mg/mL). The association of *L. pedunculata* with *S. rosmarinus* is active against 30 strains among 34.

			MIC ± SD (mg/mL)				
Microorganisms		Reference	LP	SR	Combination		FIC
					LP	SR	
Gram +	*Enterococcus faecalis*	C159-6	NA	NA	1.00 ± 0.28	0.70 ± 0.37	-
	*Enterococcus* sp.	8153	NA	NA	NA	NA	-
	*Mycobacterium smegmatis*	5003	0.43 ± 0.25	0.70 ± 0.37	0.13 ± 0.04	0.40 ± 0.14	0.87
	*Staphylococcus aureus*	8146	0.60 ± 0.00	0.60 ± 0.00	0.18 ± 0.09	0.30 ± 0.00	0.79
	*Staphylococcus aureus*	8241	0.50 ± 0.14	0.60 ± 0.00	0.15 ± 0.11	0.25 ± 0.07	0.72
	*Staphylococcus aureus*	ATCC 6538	0.60 ± 0.00	0.50 ± 0.14	0.18 ± 0.09	0.25 ± 0.07	0.79
	*Staphylococcus aureus*	T28-1	0.40 ± 0.14	0.50 ± 0.14	0.20 ± 0.07	0.13 ± 0.04	0.75
	*Staphylococcus aureus*	T17-4	0.60 ± 0.00	0.80 ± 0.28	0.08 ± 0.00	0.40 ± 0.14	0.63
	*Staphylococcus epidermidis*	T46A1	0.20 ± 0.07	0.20 ± 0.07	0.10 ± 0.04	0.10 ± 0.04	1.00
	*Staphylococcus epidermidis*	T19A1	0.25 ± 0.07	0.25 ± 0.07	0.18 ± 0.09	0.08 ± 0.00	1.00
	*Staphylococcus epidermidis*	T21A5	0.30 ± 0.00	0.25 ± 0.07	0.13 ± 0.04	0.10 ± 0.04	0.82
	*Staphylococcus warneri*	T12A12	0.30 ± 0.00	0.25 ± 0.07	0.13 ± 0.04	0.13 ± 0.04	0.92
	*Staphylococcus warneri*	T26A1	0.30 ± 0.00	0.25 ± 0.07	0.13 ± 0.04	0.13 ± 0.04	0.92
	*Staphylococcus pettenkoferi*	T47.A6	0.30 ± 0.00	0.25 ± 0.07	0.10 ± 0.04	0.13 ± 0.04	0.83
	*Streptococcus agalactiae*	T38.2	0.75 ± 0.45	0.75 ± 0.45	0.38 ± 0.23	0.60 ± 0.00	1.30
	*Streptococcus agalactiae*	T53C9	NA	1.20 ± 0.00	0.65 ± 0.43	0.50 ± 0.14	-
	*Streptococcus pyogenes*	16138	1.20 ± 0.00	1.20 ± 0.00	0.11 ± 0.04	0.38 ± 0.23	0.41
	*Streptococcus pyogenes*	16135	0.90 ± 0.30	0.60 ± 0.00	0.15 ± 0.00	0.45 ± 0.15	0.92
	*Corynebacterium striatum*	T40A3	NA	NA	0.80 ± 0.28	0.35 ± 0.19	-
Gram −	*Citrobacter freundii*	11041	NA	NA	0.80 ± 0.28	0.60 ± 0.00	-
	*Citrobacter freundii*	10268	1.20 ± 0.00	NA	0.80 ± 0.28	0.45 ± 0.21	-
	*Escherichia coli*	ATCC 25922	NA	NA	NA	NA	-
	*Escherichia coli*	T20A1	NA	NA	1.20 ± 0.00	1.20 ± 0.00	-
	*Escherichia coli*	8138	NA	NA	1.20 ± 0.00	1.20 ± 0.00	-
	*Escherichia coli*	8157	NA	NA	NA	NA	-
	*Enterobacter aerogenes*	9004	NA	NA	1.00 ± 0.28	0.70 ± 0.37	-
	*Klebsiella pneumoniae*	10270	NA	NA	1.20 ± 0.00	1.20 ± 0.00	-
	*Klebsiella pneumoniae*	11016	NA	NA	NA	NA	-
	*Proteus mirabilis*	11060	1.20 ± 0.00	1.20 ± 0.00	0.35 ± 0.19	0.33 ± 0.22	0.56
	*Proteus mirabilis*	T28-3	1.00 ± 0.28	0.60 ± 0.00	0.35 ± 0.19	0.33 ± 0.22	0.89
	*Pseudomonas aeruginosa*	8131	NA	NA	0.65 ± 0.43	0.80 ± 0.28	-
	*Pseudomonas aeruginosa*	ATCC 27583	1.20 ± 0.00	1.20 ± 0.00	0.70 ± 0.37	0.28 ± 0.23	0.81
	*Pseudomonas aeruginosa*	8129	0.50 ± 0.14	0.60 ± 0.42	0.20 ± 0.07	0.15 ± 0.11	0.65
	*Salmonella* sp.	11033	NA	NA	1.20 ± 0.00	0.80 ± 0.28	-

(NA) not active; (LP) *L. pedunculata*; (SR) *S. rosmarinus.*

**Table 5:** MIC values of *L. pedunculata* and *S. lavandulifolia* aqueous extracts individually and in combination (in mg/mL). The association of *L. pedunculata* with *S. lavandulifolia* is active against 32 strains among 33.

			MIC ± SD (mg/mL)				
Microorganisms		Reference	LP	SL	Combination		FIC
					LP	SL	
Gram +	*Enterococcus faecalis*	C159-6	NA	NA	0.10 ± 0.04	0.83 ± 0.53	-
	*Enterococcus* sp.	8153	NA	NA	0.85 ± 0.49	1.20 ± 0.00	-
	*Mycobacterium smegmatis*	5003	0.43 ± 0.25	0.50 ± 0.14	0.10 ± 0.04	0.33 ± 0.22	0.89
	*Staphylococcus aureus*	8146	0.60 ± 0.00	0.60 ± 0.00	0.08 ± 0.00	0.30 ± 0.00	0.63
	*Staphylococcus aureus*	8241	0.50 ± 0.14	0.60 ± 0.00	0.08 ± 0.00	0.30 ± 0.00	0.65
	*Staphylococcus aureus*	ATCC 6538	0.60 ± 0.00	0.50 ± 0.14	0.15 ± 0.11	0.23 ± 0.11	0.70
	*Staphylococcus aureus*	T28-1	0.40 ± 0.14	0.50 ± 0.14	0.15 ± 0.11	0.23 ± 0.11	0.83
	*Staphylococcus aureus*	T17-4	0.60 ± 0.00	0.60 ± 0.00	0.13 ± 0.04	0.30 ± 0.00	0.71
	*Staphylococcus epidermidis*	T46A1	0.20 ± 0.07	0.25 ± 0.07	0.10 ± 0.04	0.13 ± 0.04	1.00
	*Staphylococcus epidermidis*	T19A1	0.25 ± 0.07	0.25 ± 0.07	0.13 ± 0.04	0.10 ± 0.04	0.90
	*Staphylococcus epidermidis*	T21A5	0.30 ± 0.00	0.25 ± 0.07	0.10 ± 0.04	0.10 ± 0.04	0.73
	*Staphylococcus warneri*	T12A12	0.30 ± 0.00	0.25 ± 0.07	0.13 ± 0.04	0.10 ± 0.04	0.82
	*Staphylococcus warneri*	T26A1	0.30 ± 0.00	0.15 ± 0.00	0.13 ± 0.04	0.08 ± 0.00	0.92
	*Staphylococcus pettenkoferi*	T47.A6	0.30 ± 0.00	0.20 ± 0.07	0.13 ± 0.04	0.10 ± 0.04	0.92
	*Streptococcus agalactiae*	T38.2	0.75 ± 0.45	ND	ND	ND	-
	*Streptococcus agalactiae*	T53C9	NA	1.20 ± 0.00	0.18 ± 0.09	0.60 ± 0.00	-
	*Streptococcus pyogenes*	16138	1.20 ± 0.00	1.20 ± 0.00	0.20 ± 0.07	0.50 ± 0.14	0.58
	*Streptococcus pyogenes*	16135	0.90 ± 0.30	1.00 ± 0.28	0.18 ± 0.09	0.30 ± 0.21	0.49
	*Corynebacterium striatum*	T40A3	NA	NA	0.25 ± 0.25	0.90 ± 0.42	-
Gram −	*Citrobacter freundii*	11041	NA	NA	1.00 ± 0.28	0.25 ± 0.25	-
	*Citrobacter freundii*	10268	1.20 ± 0.00	NA	0.90 ± 0.42	0.28 ± 0.23	-
	*Escherichia coli*	ATCC 25922	NA	NA	1.20 ± 0.00	1.20 ± 0.00	-
	*Escherichia coli*	T20A1	NA	NA	1.00 ± 0.28	1.20 ± 0.00	-
	*Escherichia coli*	8138	NA	NA	1.20 ± 0.00	0.80 ± 0.28	-
	*Escherichia coli*	8157	NA	NA	NA	NA	-
	*Enterobacter aerogenes*	9004	NA	NA	1.20 ± 0.00	0.10 ± 0.04	-
	*Klebsiella pneumoniae*	10270	NA	NA	1.20 ± 0.00	1.20 ± 0.00	-
	*Klebsiella pneumoniae*	11016	NA	NA	1.20 ± 0.00	1.20 ± 0.00	-
	*Proteus mirabilis*	11060	1.20 ± 0.00	1.00 ± 0.28	0.20 ± 0.07	0.50 ± 0.14	0.67
	*Proteus mirabilis*	T28-3	1.00 ± 0.28	0.90 ± 0.30	0.30 ± 0.00	0.30 ± 0.00	0.63
	*Pseudomonas aeruginosa*	8131	NA	NA	0.55 ± 0.46	0.85 ± 0.49	-
	*Pseudomonas aeruginosa*	ATCC 27583	1.20 ± 0.00	1.20 ± 0.00	0.50 ± 0.14	0.60 ± 0.00	0.92
	*Pseudomonas aeruginosa*	8129	0.50 ± 0.14	0.50 ± 0.14	0.15 ± 0.00	0.15 ± 0.00	0.60
	*Salmonella* sp.	11033	NA	NA	1.20 ± 0.00	0.60 ± 0.00	-

(ND) not determined; (NA) not active; (LP) *L. pedunculata*; (SL) *S. lavandulifolia.*

**Table 6:** MIC values of *L. pedunculata* and *O. compactum* aqueous extracts individually and in combination (in mg/mL). The association of *L. pedunculata* with *O. compactum* was active against 25 strains among 34.

			MIC ± SD (mg/mL)				
Microorganisms		Reference	LP	OC	Combination		FIC
					LP	OC	
Gram +	*Enterococcus faecalis*	C159-6	NA	NA	1.20 ± 0.00	0.90 ± 0.42	-
	*Enterococcus* sp.	8153	NA	NA	NA	NA	-
	*Mycobacterium smegmatis*	5003	0.43 ± 0.25	1.00 ± 0.28	0.15 ± 0.11	0.45 ± 0.21	0.80
	*Staphylococcus aureus*	8146	0.60 ± 0.00	1.00 ± 0.28	0.15 ± 0.11	0.50 ± 0.14	0.75
	*Staphylococcus aureus*	8241	0.50 ± 0.14	0.80 ± 0.28	0.18 ± 0.09	0.23 ± 0.11	0.63
	*Staphylococcus aureus*	ATCC 6538	0.60 ± 0.00	1.00 ± 0.28	0.30 ± 0.00	0.15 ± 0.11	0.65
	*Staphylococcus aureus*	T28-1	0.40 ± 0.14	0.80 ± 0.28	0.13 ± 0.04	0.30 ± 0.00	0.69
	*Staphylococcus aureus*	T17-4	0.60 ± 0.00	0.80 ± 0.28	0.23 ± 0.11	0.25 ± 0.25	0.69
	*Staphylococcus epidermidis*	T46A1	0.20 ± 0.07	0.35 ± 0.19	0.10 ± 0.04	0.10 ± 0.04	0.79
	*Staphylococcus epidermidis*	T19A1	0.25 ± 0.07	0.40 ± 0.14	0.08 ± 0.00	0.20 ± 0.07	0.80
	*Staphylococcus epidermidis*	T21A5	0.30 ± 0.00	0.40 ± 0.14	0.08 ± 0.00	0.20 ± 0.07	0.75
	*Staphylococcus warneri*	T12A12	0.30 ± 0.00	0.50 ± 0.14	0.10 ± 0.04	0.20 ± 0.07	0.73
	*Staphylococcus warneri*	T26A1	0.30 ± 0.00	0.60 ± 0.00	0.13 ± 0.04	0.20 ± 0.07	0.75
	*Staphylococcus pettenkoferi*	T47.A6	0.30 ± 0.00	0.50 ± 0.14	0.13 ± 0.04	0.15 ± 0.00	0.72
	*Streptococcus agalactiae*	T38.2	0.75 ± 0.45	NA	0.64 ± 0.56	0.19 ± 0.11	-
	*Streptococcus agalactiae*	T53C9	NA	NA	1.00 ± 0.28	0.33 ± 0.22	-
	*Streptococcus pyogenes*	16138	1.20 ± 0.00	1.20 ± 0.00	0.45 ± 0.15	0.60 ± 0.00	0.88
	*Streptococcus pyogenes*	16135	0.90 ± 0.30	1.20 ± 0.00	0.08 ± 0.00	0.90 ± 0.30	0.83
	*Corynebacterium striatum*	T40A3	NA	NA	0.80 ± 0.28	0.45 ± 0.21	-
Gram −	*Citrobacter freundii*	11041	NA	NA	1.20 ± 0.00	0.33 ± 0.22	-
	*Citrobacter freundii*	10268	1.20 ± 0.00	NA	1.20 ± 0.00	0.23 ± 0.11	-
	*Escherichia coli*	ATCC 25922	NA	NA	NA	NA	-
	*Escherichia coli*	T20A1	NA	NA	NA	NA	-
	*Escherichia coli*	8138	NA	NA	NA	NA	-
	*Escherichia coli*	8157	NA	NA	NA	NA	-
	*Enterobacter aerogenes*	9004	NA	NA	1.20 ± 0.00	0.38 ± 0.23	-
	*Klebsiella pneumoniae*	10270	NA	NA	NA	NA	-
	*Klebsiella pneumoniae*	11016	NA	NA	NA	NA	-
	*Proteus mirabilis*	11060	1.20 ± 0.00	1.20 ± 0.00	0.60 ± 0.00	0.25 ± 0.07	0.71
	*Proteus mirabilis*	T28-3	1.00 ± 0.28	1.20 ± 0.00	0.43 ± 0.25	0.35 ± 0.19	0.72
	*Pseudomonas aeruginosa*	8131	NA	NA	NA	NA	-
	*Pseudomonas aeruginosa*	ATCC 27583	1.20 ± 0.00	NA	0.65 ± 0.43	0.70 ± 0.37	-
	*Pseudomonas aeruginosa*	8129	0.50 ± 0.14	0.80 ± 0.28	0.18 ± 0.09	0.33 ± 0.22	0.76
	*Salmonella* sp.	11033	NA	NA	NA	NA	-

(NA) not active; (LP) *L. pedunculata*; (OC) *O. compactum.*

## Data Availability

Data are available upon request.

## References

[1] Jones K.E., Patel N.G., Levy M.A., Storeygard A., Balk D., Gittleman J.L., Daszak P. (2008). Global Trends in Emerging Infectious Diseases. Nature.

[2] Alekshun M.N., Levy S.B. (2007). Molecular Mechanisms of Antibacterial Multidrug Resistance. Cell.

[3] Qi G.-B., Zhang D., Liu F.-H., Qiao Z.-Y., Wang H. (2017). An “On-Site Transformation” Strategy for Treatment of Bacterial Infection. Adv. Mater..

[4] Levin A.S., Barone A.A., Penço J., Santos M.V., Marinho I.S., Arruda E.A., Manrique E.I., Costa S.F. (1999). Intravenous Colistin as Therapy for Nosocomial Infections Caused by Multidrug-Resistant *Pseudomonas Aeruginosa* and *Acinetobacter Baumannii*. Clin. Infect. Dis. Off. Publ. Infect. Dis. Soc. Am..

[5] Blair J.M.A., Webber M.A., Baylay A.J., Ogbolu D.O., Piddock L.J.V. (2015). Molecular Mechanisms of Antibiotic Resistance. Nat. Rev. Microbiol..

[6] Ghobadi A., Amini-Behbahani F., Yousefi A., Shirazi M.T., Behnoud N. (2019). Medicinal and Nutritional Properties of *Ziziphus Jujuba* Mill. in Traditional Persian Medicine and Modern Phytotherapy. Crescent J. Med. Biol. Sci..

[7] Mickymaray S. (2019). Efficacy and Mechanism of Traditional Medicinal Plants and Bioactive Compounds against Clinically Important Pathogens. Antibiotics.

[8] Baba D. (2015). Stratégies Nationale de Développement Des Plantes Aromatiques et Médicinales Spontannées.

[9] Erland L.A.E., Mahmoud S.S., Preedy V.R. (2016). Chapter 57—Lavender (*Lavandula Angustifolia*) Oils. Essential Oils in Food Preservation, Flavor and Safety.

[10] Nafis A., Ouedrhiri W., Iriti M., Mezrioui N., Marraiki N., Elgorban A.M., Syed A., Hassani L. (2021). Chemical Composition and Synergistic Effect of Three Moroccan Lavender EOs with Ciprofloxacin against Foodborne Bacteria: A Promising Approach to Modulate Antimicrobial Resistance. Lett. Appl. Microbiol..

[11] Ouhaddou H., Boubaker H., Msanda F., El Mousadik A. (2015). An Ethnobotanical Study of Medicinal Plants of the Agadir Ida Ou Tanane Province (Southwest Morocco). J. Appl. Biosci..

[12] Teixidor-Toneu I., Martin G.J., Ouhammou A., Puri R.K., Hawkins J.A. (2016). An Ethnomedicinal Survey of a Tashelhit-Speaking Community in the High Atlas, Morocco. J. Ethnopharmacol..

[13] Almohawes Z.N., Alruhaimi H.S. (2019). Effect of *Lavandula Dentata* Extract on Ovalbumin-Induced Asthma in Male Guinea Pigs. Braz. J. Biol..

[14] Baptista R., Madureira A.M., Jorge R., Adão R., Duarte A., Duarte N., Lopes M.M., Teixeira G. (2015). Antioxidant and Antimycotic Activities of Two Native *Lavandula* Species from Portugal. Evid. Based Complement. Alternat. Med..

[15] Kulabas S.S., Ipek H., Tufekci A.R., Arslan S., Demirtas I., Ekren R., Sezerman U., Tumer T.B. (2018). Ameliorative Potential of *Lavandula Stoechas* in Metabolic Syndrome via Multitarget Interactions. J. Ethnopharmacol..

[16] Bint Mustafa S., Akram M., Muhammad Asif H., Qayyum I., Mehmood Hashmi A., Munir N., Said Khan F., Riaz M., Ahmad S. (2019). Antihyperglycemic Activity of Hydroalcoholic Extracts of Selective Medicinal Plants *Curcuma Longa*, *Lavandula Stoechas*, *Aegle Marmelos*, and *Glycyrrhiza Glabra* and Their Polyherbal Preparation in Alloxan-Induced Diabetic Mice. Dose-Response Int. J..

[17] Rahmati B., Kiasalari Z., Roghani M., Khalili M., Ansari F. (2017). Antidepressant and Anxiolytic Activity of *Lavandula Officinalis* Aerial Parts Hydroalcoholic Extract in Scopolamine-Treated Rats. Pharm. Biol..

[18] Sadraei H., Asghari G., Rahmati M. (2019). Study of Antispasmodic Action of *Lavandula Angustifolia* Mill Hydroalcoholic Extract on Rat Ileum. J. Herbmed Pharmacol..

[19] Shahdadi H., Bahador R.S., Eteghadi A., Boraiinejad S. (2017). Lavender a Plant for Medical Uses: A Literature Review. Indian J. Public Health Res. Dev..

[20] Boutahiri S., Bouhrim M., Abidi C., Mechchate H., Alqahtani A.S., Noman O.M., Kouoh Elombo F., Gressier B., Sahpaz S., Bnouham M. (2021). Antihyperglycemic Effect of *Lavandula Pedunculata*: In Vivo, In Vitro and Ex Vivo Approaches. Pharmaceutics.

[21] Chaachouay N., Benkhnigue O., Fadli M., El Ibaoui H., El Ayadi R., Zidane L. (2019). Ethnobotanical and Ethnopharmacological Study of Medicinal and Aromatic Plants Used in the Treatment of Respiratory System Disorders in the Moroccan Rif. Ethnobot. Res. Appl..

[22] Zougagh S., Belghiti A., Rochd T., Zerdani I., Mouslim J. (2019). Medicinal and Aromatic Plants Used in Traditional Treatment of the Oral Pathology: The Ethnobotanical Survey in the Economic Capital Casablanca, Morocco (North Africa). Nat. Prod. Bioprospecting.

[23] Zeroual A., Eloutassi N., Chaouch M., Chaqroune A. (2020). Antimicrobial, Antioxidant Activity, and Chemical Composition of *Origanum Compactum* Benth from Taounate Province, North Morocco. Asian J. Pharm. Clin. Res..

[24] Elansary H.O., Szopa A., Kubica P., Ekiert H., El-Ansary D.O., Al-Mana F.A., Mahmoud E.A. (2020). Saudi *Rosmarinus Officinalis* and *Ocimum Basilicum* L. Polyphenols and Biological Activities. Processes.

[25] Pop (Cuceu) A.V., Tofană M., Socaci S.A., Pop C., Rotar A.M., Nagy M., Salanţă L. (2016). Determination of Antioxidant Capacity and Antimicrobial Activity of Selected *Salvia* Species. Bull. Univ. Agric. Sci. Vet. Med. Cluj-Napoca Food Sci. Technol..

[26] Lopes C.L., Pereira E., Soković M., Carvalho A.M., Barata A.M., Lopes V., Rocha F., Calhelha R.C., Barros L., Ferreira I.C.F.R. (2018). Phenolic Composition and Bioactivity of *Lavandula Pedunculata* (Mill.) Cav. Samples from Different Geographical Origin. Molecules.

[27] Zhang Q., Zhang J., Shen J., Silva A., Dennis D.A., Barrow C.J. (2006). A Simple 96-Well Microplate Method for Estimation of Total Polyphenol Content in Seaweeds. J. Appl. Phycol..

[28] Olounlade P.A., Hounzangbe-Adote M.S., Azando E.V.B., Ha T.T., Brunet S., Moulis C., Fabre N., Fouraste I., Hoste H., Valentin A. (2011). Etude in vitro de l’effet Des Tanins de *Newbouldia Laevis* et de *Zanthoxylum Zanthoxyloïdes* Sur La Migration Des Larves Infestantes de *Haemonchus Contortus*. Int. J. Biol. Chem. Sci..

[29] CLSI (2012). Methods for Dilution Antimicrobial Susceptibility Tests for Bacteria That Grow Aerobically; Approved Standard.

[30] CLSI (2020). Performance Standards for Antimicrobial Susceptibility Testing.

[31] Fratini F., Mancini S., Turchi B., Friscia E., Pistelli L., Giusti G., Cerri D. (2017). A Novel Interpretation of the Fractional Inhibitory Concentration Index: The Case *Origanum Vulgare* L. and *Leptospermum Scoparium* J. R. et G. Forst Essential Oils against *Staphylococcus Aureus* Strains. Microbiol. Res..

[32] Blažeković B., Yang W., Wang Y., Li C., Kindl M., Pepeljnjak S., Vladimir-Knežević S. (2018). Chemical Composition, Antimicrobial and Antioxidant Activities of Essential Oils of *Lavandula* × *Intermedia* ‘Budrovka’ and *L. Angustifolia* Cultivated in Croatia. Ind. Crops Prod..

[33] Moussi Imane M., Houda F., Said Amal A.H., Kaotar N., Mohammed T., Imane R., Farid H. (2017). Phytochemical Composition and Antibacterial Activity of Moroccan *Lavandula Angustifolia* Mill. J. Essent. Oil Bear. Plants.

[34] Barkaoui M., Katiri A., Boubaker H., Msanda F. (2017). Ethnobotanical Survey of Medicinal Plants Used in the Traditional Treatment of Diabetes in Chtouka Ait Baha and Tiznit (Western Anti-Atlas), Morocco. J. Ethnopharmacol..

[35] Orch H., Douira A., Zidane L. (2015). Étude Ethnobotanique Des Plantes Médicinales Utilisées Dans Le Traitement Du Diabète, et Des Maladies Cardiaques Dans La Région d’Izarène (Nord Du Maroc). J. Appl. Biosci..

[36] Zaher A., Boufellous M., Jaber H., Hartiti H.E., Barrahi M., Ouhssine M., Bourkhiss B. (2018). Ethnobotanical Study of Medicinal Plants Used in the Province of Sidi Slimane (Morocco). J. Biosci. Med..

[37] Horky P., Skalickova S., Smerkova K., Skladanka J. (2019). Essential Oils as a Feed Additives: Pharmacokinetics and Potential Toxicity in Monogastric Animals. Animals.

[38] Ramdan B., El Malki F., Eddarraji K., Greche H., Nhiri M. (2018). Composition and Antibacterial Activity of Hydro-Alcohol and Aqueous Extracts Obtained from the Lamiaceae Family. Pharmacogn. J..

[39] Giner M.J., Vegara S., Funes L., Martí N., Saura D., Micol V., Valero M. (2012). Antimicrobial Activity of Food-Compatible Plant Extracts and Chitosan against Naturally Occurring Micro-Organisms in Tomato Juice. J. Sci. Food Agric..

[40] Casciaro B., Calcaterra A., Cappiello F., Mori M., Loffredo M.R., Ghirga F., Mangoni M.L., Botta B., Quaglio D. (2019). Nigritanine as a New Potential Antimicrobial Alkaloid for the Treatment of *Staphylococcus Aureus*-Induced Infections. Toxins.

[41] Kourtis A.P., Hatfield K., Baggs J., Mu Y., See I., Epson E., Nadle J., Kainer M.A., Dumyati G., Petit S. (2019). Vital Signs: Epidemiology and Recent Trends in Methicillin-Resistant and in Methicillin-Susceptible *Staphylococcus Aureus* Bloodstream Infections—United States. Morb. Mortal. Wkly. Rep..

[42] Wang Y.-Z., Fu S.-G., Wang S.-Y., Yang D.-J., Wu Y.-H.S., Chen Y.-C. (2018). Effects of a Natural Antioxidant, Polyphenol-Rich Rosemary (*Rosmarinus Officinalis* L.) Extract, on Lipid Stability of Plant-Derived Omega-3 Fatty-Acid Rich Oil. LWT Food Sci. Technol..

[43] Bourhia M., Laasri F.E., Aourik H., Boukhris A., Ullah R., Bari A., Ali S.S., El Mzibri M., Benbacer L., Gmouh S. (2019). Antioxidant and Antiproliferative Activities of Bioactive Compounds Contained in *Rosmarinus Officinalis* Used in the Mediterranean Diet. Evid. Based Complement. Alternat. Med..

[44] Afonso A.F., Pereira O.R., Fernandes Â., Calhelha R.C., Silva A.M.S., Ferreira I.C.F.R., Cardoso S.M. (2019). Phytochemical Composition and Bioactive Effects of *Salvia Africana*, *Salvia Officinalis* ‘Icterina’ and *Salvia Mexicana* Aqueous Extracts. Molecules.

[45] Bouyahya A., Abrini J., Bakri Y., Dakka N. (2017). Screening phytochimique et évaluation de l’activité antioxydante et antibactérienne des extraits d’*Origanum compactum*. Phytothérapie.

[46] Dong G., Liu H., Yu X., Zhang X., Lu H., Zhou T., Cao J. (2018). Antimicrobial and Anti-Biofilm Activity of Tannic Acid against *Staphylococcus Aureus*. Nat. Prod. Res..

[47] Štumpf S., Hostnik G., Primožič M., Leitgeb M., Salminen J.-P., Bren U. (2020). The Effect of Growth Medium Strength on Minimum Inhibitory Concentrations of Tannins and Tannin Extracts against *E. coli*. Molecules.

[48] Nadeem M., Imran M., Aslam Gondal T., Imran A., Shahbaz M., Muhammad Amir R., Wasim Sajid M., Batool Qaisrani T., Atif M., Hussain G. (2019). Therapeutic Potential of Rosmarinic Acid: A Comprehensive Review. Appl. Sci..

[49] Matejczyk M., Świsłocka R., Golonko A., Lewandowski W., Hawrylik E. (2018). Cytotoxic, Genotoxic and Antimicrobial Activity of Caffeic and Rosmarinic Acids and Their Lithium, Sodium and Potassium Salts as Potential Anticancer Compounds. Adv. Med. Sci..

[50] Mohammed E.T., Mustafa Y.F. (2020). Coumarins from Red Delicious Apple Seeds: Extraction, Phytochemical Analysis, and Evaluation as Antimicrobial Agents. Syst. Rev. Pharm..

[51] Wang M., Firrman J., Liu L., Yam K. (2019). A Review on Flavonoid Apigenin: Dietary Intake, ADME, Antimicrobial Effects, and Interactions with Human Gut Microbiota. BioMed Res. Int..

[52] Costa P., Gonçalves S., Valentão P., Andrade P.B., Almeida C., Nogueira J.M.F., Romano A. (2013). Metabolic Profile and Biological Activities of *Lavandula Pedunculata* Subsp. *Lusitanica* (Chaytor) Franco: Studies on the Essential Oil and Polar Extracts. Food Chem..

[53] Barbieri J.B., Goltz C., Batistão Cavalheiro F., Theodoro Toci A., Igarashi-Mafra L., Mafra M.R. (2020). Deep Eutectic Solvents Applied in the Extraction and Stabilization of Rosemary (*Rosmarinus Officinalis* L.) Phenolic Compounds. Ind. Crops Prod..

[54] Lu Y., Yeap Foo L. (2002). Polyphenolics of *Salvia*—A Review. Phytochemistry.

